# Social values for health technology assessment in Canada: a scoping review of hepatitis C screening, diagnosis and treatment

**DOI:** 10.1186/s12889-020-8190-2

**Published:** 2020-01-20

**Authors:** Caroline O’Keefe-Markman, Kristina Dawn Lea, Christopher McCabe, Elaine Hyshka, Tania Bubela

**Affiliations:** 1 School of Public Health, University of Alberta, Edmonton, AB Canada; 2 Faculty of Medicine and Dentistry, University of Alberta, Edmonton, AB Canada; 3 Institute of Health Economics, Edmonton, AB Canada; 4 Department of Emergency Medicine, Faculty of Medicine and Dentistry, University of Alberta, Edmonton, AB Canada; 5 Inner City Health and Wellness, Royal Alexandra Hospital, Edmonton, AB Canada; 6 Faculty of Health Sciences, Simon Fraser University, Burnaby, BC Canada

**Keywords:** Health technology assessment, Social values, Hepatitis C, Screening, Diagnosis, Direct acting antivirals

## Abstract

**Background:**

Health care system decision makers face challenges in allocating resources for screening, diagnosis and treatment of hepatitis C. Approximately 240,000 individuals are infected with the hepatitis C virus (HCV) in Canada. Populations most affected by HCV include Indigenous people, people who inject drugs, immigrants and homeless or incarcerated populations as well as those born between 1946 and 1965. Curative but expensive drug regimens of novel direct acting antivirals (DAAs) are available. We aim to identify social values from academic literature for inclusion in health technology assessments.

**Methods:**

We conducted a scoping review of academic literature to identify and analyze the social values and evidence-based recommendations for screening, diagnosis and treatment of HCV in Canada. After applying inclusion/exclusion criteria, we abstracted: type of intervention(s), population(s) affected, study location, screening methods, diagnostics and treatments. We then abstracted and applied qualitative codes for social values. We extracted social value statements and clustered them into one of 4 categories: (1) equity and justice, (2) duty to provide care, (3) maximization of population benefit, and (4) individual versus community interests.

**Results:**

One hundred and eighteen articles met our inclusion criteria on screening, diagnosis and treatment of HCV in Canada. Of these, 54 (45.8%) discussed screening, 4 (3.4%) discussed diagnosis and 60 (50.8%) discussed treatment options. Most articles discussed the general population and other non-vulnerable populations. Articles that discussed vulnerable populations focused on people who inject drugs. We coded 1243 statements, most of which fell into the social value categories of equity and justice, duty to provide care and maximization of population benefit.

**Conclusion:**

The academic literature identified an expanded set of social values to be taken into account by resource allocation decision makers in financially constrained environments. In the context of hepatitis C, authors called for greater consideration of equity and justice and the duty to provide care in making evidence-based recommendations for screening, diagnosis and treatment for different populations and in different settings that also account for individual and community interests.

## Background

Health Technology Assessment (HTA) agencies make recommendations for resource allocation decision making in Canada’s publicly funded health care systems informed by cost effectiveness analyses. Technologies considered by HTA agencies include screening programs, diagnostic tests and therapeutics. HTA is often characterized as considering efficiency, defined as the balance that maximizes population health outcomes for given resources [1]. As such, it has been criticized as being overly utilitarian in approach. Decisions premised in utilitarianism maximize wellbeing and limit the loss of wellbeing for individuals; the most successful outcomes increase wellbeing with minimal corresponding loss [1, 2]. However, such approaches may fail to consider other measures for an acceptable amount of loss and benefit, including consideration of a broader set of social values [1–3]. Here, we aim to identify the social values from the academic literature that could be included in HTA decision making for the screening, diagnosis and treatment of an exemplar infectious disease – hepatitis C.

In most Western countries, ageing populations, combined with expensive, innovative therapies, raise health care expenditures and strain health care budgets. This confluence of factors drives the application of explicit criteria on who can access treatments and when [4]. The cost of novel treatments has called into question the feasibly of providing access to all for whom they are clinically indicated. This in turn has led to demands for formal, transparent and ethical review processes of new health care technologies [4] that consider a broader set of social values [5–8].

Some HTA agencies are responding to the challenge of integrating social values into their analyses by consulting with publics about the social values and population characteristics that ought to be taken into account by decision makers when making resource allocation decisions. For example, the Canadian Agency for Drugs and Technologies in Health (CADTH), when considering the cost effectiveness of screening programs, additionally takes into account patient preferences [5]. Indeed, CADTH aims to incorporate a broader set of values into its decision-making framework, including the patient perspective [9]. There are many methods for eliciting information on patient and social values. For example, a citizens’ jury, comprised of members of the public is one mechanism to incorporate diverse perspectives. An expert panel presents the jury with information on health technology innovations. Jurors are then asked to complete questionnaires, explicating HTA priorities [5]. This process helps guide resource allocation decision-making that includes the adoption of new therapies [5]. Another method is discreet choice analysis, which also places members of the public at the forefront of decision-making and enables elucidation of values that may then be incorporated into resource allocation choices [10]. Although these mechanisms try to elicit social values that might be incorporated into the decision making process [11] and add to transparency in decision making, there are practical and philosophical challenges involved in this type of work. These include deciding what should be valued, whose values should be included, how to make trade-offs between equity and efficiency, and, how quantitative equity weights should be obtained [5, 6].

In this paper we focus on the question of what should be valued. To address this question, we undertook a scoping review of the published academic literature in relation to screening, diagnosis and treatment of Hepatitis C virus (HCV) in Canada. Our analysis is timely because current policy debates about the use of novel, effective but costly direct acting antivirals (DAAs) for the hepatitis C virus (HCV) are most pronounced for marginalized populations, which necessitates different approaches to their care [12, 13]. While issues of prevention of HCV infection are important, a discussion of prevention programs is beyond the scope of our analysis. The exception is “treatment as prevention” whereby effective treatment of an infectious disease, such as HIV and HCV, at scale may prevent further infections [14, 15].

In the context of hepatitis C, social values that might augment HTA include equity in health outcomes, defined as the absence of socially unjust or unfair health disparities [16] and justice, defined as fair, equitable and appropriate treatment in light of what is owed or due to persons [17]. Considerations of justice enable a more complete representation of the contextual factors and social determinants of health that affect those living with hepatitis C. Values such as equity and justice may be particularly relevant when populations under consideration are marginalized [13]. In a resource-constrained environment, consideration of such social values is paramount to ensure a just distribution of resources that considers more than cost alone.

### Hepatitis C screening, diagnosis, and treatment in Canada

Novel but costly direct acting antivirals (DAAs), namely, Epclusa ($74,760 CAD), Sovaldi ($84,000), Harvoni ($95,000), Holkira Pak ($55,860), Zepatier ($60,300), Sunvepra ($89,000), Daklinza ($95,550), Technivie ($58,656) and Galexos ($96,078) (all costs quoted in Canadian Dollars) have fewer adverse effects on patients than the previous standard of care, interferon based treatments [18–20]. They have a cure rate of > 90% with an 8 to 12-week regimen [20]. They require a once daily tablet instead of the complex treatment regimens of older HCV drugs. Although there have been major medical advances in the treatment for HCV, these drugs remain expensive. The large budget impact stemming for the combination of expensive therapies and high disease prevalence (220,697 to 245,987 Canadians) has resulted in limits being placed on who can receive treatment within the licensed indications [18].

The high cost of DAAs has limited the populations in which they are employed. For example, in the Province of Alberta, treatment is available to those who have a fibrosis score (liver stiffness score and progression of disease) of F2 or above, signifying moderate liver fibrosis prior to being given access to curative treatment [21]. Exceptions are made for persons co-infected with HIV or hepatitis B virus (because of the greatest risk of liver disease in those patients), co-existent liver disease with evidence of fatty liver disease, post organ transplant, extra-hepatic manifestations, chronic kidney disease, diabetes, and woman of childbearing age planning pregnancy within the next 12 months [21]. This decision is based mostly on cost but it has been argued fails to consider the larger population health effect of treating high-risk populations as a means of preventing transmission [13].

Treatment advances have led to revised recommendations on screening for HCV [12], for example, by the Canadian Task Force on Preventive Health Care [21]. The Task Force uses a Grading of Recommendations Assessment, Development and Evaluation system (GRADE), based on the effectiveness of screening in different populations and cost [22]. Each population receives a GRADE of strong or weak. Strong suggests both a high level of evidence in support of screening in a particular population and high desirability of outcomes. Weak suggests the inverse [22]. Based on GRADE, the Task Force recommended screening for people who have injected drugs, those who have been incarcerated, and individuals who received blood transfusions prior to 1992. It did not recommend screening for the general public or the baby boomer cohort [22], as the United States (US) Center for Disease Control (CDC) has done. The Task Force might have reached different conclusions if it had considered a broader range of social values, such as those we identified.

## Methods

We conducted a scoping review of the literature to identify and analyze the social values and evidence-based recommendations for screening, diagnosis and treatment of HCV in Canada. Following the methodology of Arksey and O’Malley’s (2005), we collected, organized and included articles in our scoping review based on a search strategy and inclusion/exclusion criteria developed in consultation with experts [23]. From included articles, we abstracted: type of intervention(s), population(s) affected, location of study, screening methods, diagnostics and treatment options. We then abstracted qualitative codes for ethical considerations and social values (Table 1). Specifically, we coded each article for social values (Table 1). We extracted social value statements and then clustered them into one of 4 categories: [1] equity and justice, [2] duty to provide care, [3] maximization of population benefit, and [4] individual versus community interests. Specifically, we followed the following 5 steps.

**Table 1:** Definitions of social value codes clustered by category

Category	Social Values Incorporated	Definition
Equity and Justice	Equity	Absence of socially unjust or unfair health disparities [16]
	Inequity	Differences in health that are unjust, unfair, unnecessary and avoidable [16]
	Justice	Fair, equitable and appropriate treatment in light of what is owed or due to persons [17]
	Distributive Justice	Persons in like need ought to be treated the same way [4]
	Egalitarianism	All humans are equal and should be afforded equal rights and opportunities [24]
Duty to Provide Care	Portability	Requires provinces to cover insured health services provided to their residents while they are temporarily absent from their province of residence or from Canada [25]
	Accessibility	Insured persons must have reasonable and uniform access to insured health services, free of financial or other barriers; No-one may be discriminated against on the basis of such factors as income, age and health status [25]
	Publicly Administered	Each provincial health care insurance plan must be administered on a non-profit basis by a public authority [25]
	Universality	Demands that all residents in the province have access to public health care insurance and insured services on uniform terms and conditions [25]
	Reciprocity	Society must be prepared to facilitate individuals and communities in their efforts to discharge their duties, i.e., public health agencies should assist individuals in complying with health measures [17]
	Duty to Provide Care	Obligation to provide safe, competent and ethical care [24]
Maximization of Population Benefit	Efficiency	The balance that maximizes outcomes for given resources [1]
	Utilitarianism	The best action is the one that maximizes the well-being of all sentient beings. Supremacy to the needs of the community as it will benefit the largest number of individuals [26]
Individual Vs. Community Interests	Liberalism	Right of an individual to pursue their own conception of good (defined as beliefs about what makes life valuable or worthwhile) [27]
	Libetarianism	People should have freedom and autonomy of choice so long as it does not interfere with others autonomy and freedoms [28]
	Welfarism	Individual preferences, desires and decisions are the most important factors when doing an economic analysis [1]
	Autonomy	The right for an individual to make his or her own choice [17]
	Communitarianism	Emphasizes the responsibility of the individual to the community [24] Community should be at the forefront of our moral thinking [27]
	Consequentialism	The consequences of an action serve as the judgment of the rightness or wrongness of the action [29]

### Step 1: development of research question

We consulted with infectious diseases, HTA and ethics experts to identify the research question in the context of resource constraints facing Canadian health systems and the market authorization for screening, diagnosis and curative novel therapies for HCV in Canada. In reviewing the literature, our research question was: What social values are implied in recommendations on the populations that should receive access to screening, diagnosis and treatment for HCV? Our analysis will inform decision makers about the social values identified from the academic literature on hepatitis C and HTA in Canada that might be taken into account when making resource allocation decisions.

### Step 2: literature search

**Fig. 1:**
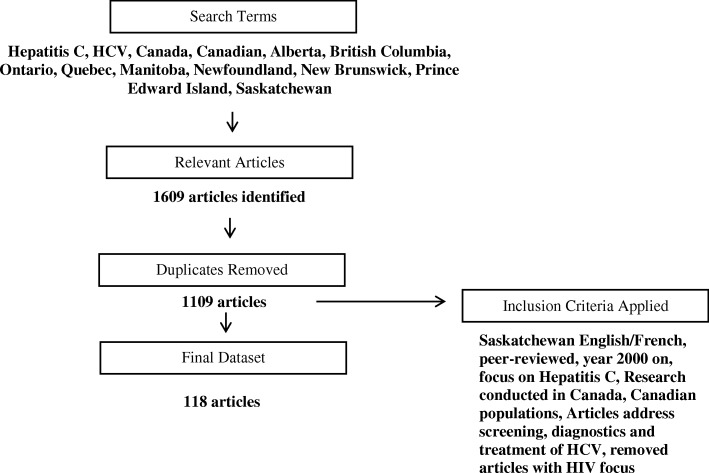
PRISMA flowchart of final study inclusion

We consulted a health sciences librarian to develop a list of key words and journals of interest. Search terms included synonyms for HCV or hepatitis C, combined with synonyms for screening, diagnosis and treatment (Fig. 1). We performed a literature search in OVID. OVID is a search engine that simultaneously searches multiple databases, covering the medical, policy, economics and HTA literature. The databases searched were: EMBASE, MEDLINE, NHS Economic Evaluation Database and Health Technology Assessment with published dates ranging from 2000- February 1st 2016.

### Step 3: application of inclusion/exclusion criteria

Two coders (COM and KGL) applied the inclusion/exclusion criteria to the articles that met the search criteria and removed duplicates. We included articles that were in English or French, described research conducted in Canada or on a Canadian population and whose focus was hepatitis C. In other words, while the study authors may not have been Canadian, all study populations were Canadian. We excluded articles that described basic science experiments, described drug mechanisms of action, had a focus on HIV (Human Immunodeficiency Virus), only addressed prevention of HCV or were published before 2000. Two coders independently coded the entire subset of articles. Each coder received training and disagreements were resolved during the training stage before coding independently. We calculated the kappa coefficient in Microsoft Excel as 0.85 as a means of determining inter-coder reliability.

### Step 4: descriptive analysis of included articles

We developed an online form to code each article for: study population characteristics (people who inject drugs (PWID), Baby boomers (those born between 1945 and 1965), indigenous peoples, individuals who received blood transfusions prior to 1992, high risk youth, prison inmates and general patient population); and location of the study (clinic, urban, prison, inner city, rural, and community health center); as well as the intervention type (screening, diagnosis, and treatment). Individuals who received blood transfusions prior to 1992 were included because a sensitive screening test for Hepatits C did not became available until 1992, and this population is recommended for screening in Canada and by the United States Centers for Disease Control and Prevention [22].

### Step 5: qualitative analysis of social values articulated in articles

We imported all full text articles into NVIVO for analysis. Our analysis used an a priori coding scheme (Table 1) that we developed based on literature on biomedical and public health ethics and social values, including Childress and Beauchamp [17], the Canada Health Act [25], Culyer and Cookson [1], and Singer [30]. Drawing on these works, we defined key social values and verified these in consultation with experts in infectious diseases, HTA and ethics. We merged codes based on these discussions to more clearly represent social value statements. We then clustered social value codes into four categories: (1) equity and justice; (2) duty to provide care; (3) maximization of population benefit (4) individual versus community interests (Table 1). We then coded the articles that met out inclusion/exclusion criteria. In the early stages of our analysis, COM and KGL independently coded 20% of the articles, and discussed any discrepancies in the coding. Each coder received training and disagreements were resolved during the training stage before coding independently. The two coders reached consensus on these articles and then coded the remaining articles independently. No new codes emerged and the a priori code book was implemented accurately. We calculated the kappa coefficient in Microsoft Excel to determine inter-coder reliability.

## Results

The number of articles that met our inclusion criteria on screening, diagnosis and treatment of HCV in a Canadian context was 118 (Fig. 1). Of these, 54 (45.8%) discussed screening, 4 (3.4%) discussed diagnosis and 60 (50.8%) discussed treatment options. Most articles considered the general population and other non-vulnerable populations, i.e., baby boomers and individuals who received blood transfusions prior to 1992.

Articles that discussed vulnerable populations focused on PWID, but few considered other vulnerable populations such as at-risk youth, prison inmates, and indigenous peoples (Table 2). Most articles did not specify the study location. Of those that did specify location, most were conducted in clinics, but few were conducted in other locations, such as rural locations and community health centers.

**Table 2:** General characteristics of populations and study locations discussed in 118 articles on screening, diagnostics, and treatment of hepatitis C in Canada

Population Characteristic			Number of Articles^a^	Percentage of Articles
Type of Population	Vulnerable Populations	Persons who use injection drugs (PWID)	35	29.6
		At-risk youth	8	6.7
		Prison populations	7	5.9
		Indigenous people	7	5.9
	Non-vulnerable populations	Baby boomers^b^	11	9.3
		Blood transfusion recipients^c^	11	9.3
		General patient population	39	33.1
Study Location	General Location	Inner City	12	10.2
		Urban	8	6.8
		Rural	4	3.4
	Specific Study Location	Clinic	18	15.3
		Prison	10	8.5
		Community health centre	5	4.2
		Not specified	61	51.7

^a^ Articles could discuss more than one type of population or location

^b^ those born between 1945 and 1965

^c^ prior to 1992

### Analysis of social values

The social value categories of equity and justice, duty to provide care and maximization of population benefit occurred most frequently in the 118 articles (Table 3). In total, we coded 1243 statements, some of which were double coded within the 118 articles. Our kappa coefficient for inter-coder reliability of 0.96 indicated excellent agreement. Most statements fell within the category of the duty to provide care (Table 3) and were focused on screening and treatment.

**Table 3:** Social value categories in 118 articles on screening, diagnosis, and treatment of HCV in Canada

Social Value Category	Number of Articles	% of Articles (*n* = 118)	Number of Coded Statements^a^	% of Coded Statements (*n* = 1243)
Equity and Justice	78	66.1	388	31.2
Duty to Provide Care	76	64.4	412	33.1
Maximization of Population Benefit	71	60.1	350	28.1
Individual vs Community Interests	27	23.0	93	7.5

^a^ Note that articles could contain statements in more than one social value category

Only 4 articles discussed diagnosis.

Most coded statements on screening fell in the category of equity and justice (*n* = 242) as did most coded statements that referenced treatment (*n* = 220).

Articles on treatment discussed modes of provision, the populations most in need of treatment and treatment guidelines. Other coded statements on treatment fell within the categories of maximization of population benefit (*n* = 172) and equity and justice (*n* = 175), with reference to high risk but vulnerable populations, such as PWID, who have inequitable access to treatment (*n* = 157). Duty to provide care was the most common category for statements about PWID (*n* = 195) and at-risk youth (*n* = 27), while maximization of population benefit was the most common category for statements about prison inmates (n = 27) and baby boomers (*n* = 61). Statements about blood transfusion recipients (*n* = 4) and indigenous peoples (*n* = 22) most commonly fell in the category of equity and justice.

Only 57 of the 118 articles specified a study location, most commonly clinics and inner-city locales (Table 2). Only four articles described research in a rural setting. Nevertheless, some differences were apparent in social value statements. Most statements in clinic- (*n* = 85) and inner city- (*n* = 75) based articles (n = 75) fell in the category of duty to provide care, while statements in urban- (*n* = 30) and community health care- (*n* = 50) based articles fell in the category of individual versus community interests. Most statements in prison-based studies fell in categories of equity and justice (*n* = 82) or duty to provide care (*n* = 73).

### Examples of social value codes

In this section, we provide excerpts from the literature as examples of our coding of the social values statements (Table 1).

#### Equity and justice

The category of equity and justice included codes for equity, justice, distributive justice and egalitarianism. Equity refers to an absence of socially unjust or unfair health disparities [16]. Inequities arise when there are differences in health that are unjust, unfair, unnecessary and/or avoidable [16]. For example: “Aboriginal people are not only disproportionately represented among HCV infected people in Canada but also underrepresented in community based treatment programs” [31].

Articles made justice claims in reference to (a) individuals who had been infected with HCV through no fault of their own, following an unscreened blood transfusion; (b) indigenous populations due to historical injustices, including trauma as a result of the residential school system and (c) prisoners who cannot freely access health care while incarcerated. Exemplar statements include:

*Blood Transfusion Recipients*: “The difficulties involved in all forms of HCV look back emphasize the importance of informing patients and their families that they have received transfusion therapy that carries certain risks” [32].

*Indigenous Peoples*: “The findings confirm the necessity of acknowledging the role of historic trauma in the health of Aboriginal peoples…. The Public Health Agency of Canada estimates that the prevalence of HCV infection is 0.8 percent in the general population in Canada and seven fold higher among Aboriginal people” [33].

*Prison Populations*: “Given the dire conditions in federal prisons today, our federal government should respond with a sensible approach to drug policy based on solid scientific evidence, sound public health principles and respect for human rights- both inside and outside of prison…” [34].

Distributive justice expands on justice by stating that persons in like need ought to be treated the same way [1]. For example, “[w]ith new medications that cure over 90% of hepatitis C, liver disease experts are urging that screening recommendations be expanded to include all Canadians born between 1945 and 1975” [35]. Under the code for egalitarianism, all humans are considered equal and should be afforded equal rights and opportunities [24]. For example: “[t]he decision to treat HCV infected persons should be considered on a case by case basis and should not necessarily exclude persons based on their use of illicit drugs” [36].

#### Duty to provide care

Duty to provide care encompasses the values articulated in the Canada Health Act (CHA), namely: accessibility, public administration, comprehensiveness, portability and universality. Of these, articles referenced the values of accessibility, comprehensiveness, and universality but not portability (coverage rules for Canadians who move between provinces) and public administration (provincial insurance plans must be administered on a non-profit basis by a public authority). In addition, in putting the CHA into practice, provinces comply with the ethical principle of reciprocity [17]. For example, “[b]ecause most new HCV infections occur as a result of IDU (injection drug use)…Clinicians may need to alter their guidelines and emphasis to reach vulnerable populations that are disproportionately affected by HCV and HIV” [37].

Accessibility refers to insured persons having reasonable and uniform access to insured health services, free of financial or other barriers. Individuals may not be discriminated against on the basis of such factors as income, age and health status [25]:

“Access to specialists in Canada via health care professional referral may be a barrier to HCV care. However, clinics that operate in conjunction with the hepatitis C Program, Edmonton Alberta, allow self-referral. It is hypothesized that this improves access to care without increasing inappropriate referrals” [38].

Comprehensiveness states that provincial health care insurance plans must all include services that are medically necessary, including hospitalization and doctors, however most plans do not cover the cost of outpatient medications. “Current programs and services are marked by inconsistent implementation and accessibility, both within individual institutions and across the federal prison system as a whole” [39].

Universality demands that all residents in the province have access to public health care insurance and insured services on uniform terms and conditions [25]:

“Treating HCV in the prison population is no less likely to fail than treating this disease in the community at large, and that the closer monitoring of psychiatric side effects in prison setting allows interferon to be safely administered even in inmates with a previous history of psychiatric illness” [40].

#### Maximization of population benefit

Maximization of population benefit combines the social values of utilitarianism and efficiency. Under utilitarianism, the best action is the one that maximizes the well-being of all sentient beings. Utilitarianism privileges the needs of the community and aims to derive benefits for the greatest number of individuals [26]. Treatment of the group (previously infected inmates) is highlighted as a means of benefiting the broader community and decreasing the overall burden of disease. For example:

“Because offenders may enter the correctional system already infected, correctional health care assumes the responsibility for caring for those infected and preventing the transmission of disease infected inmates. With most offenders eventually returning to the community, the correctional setting also represents a critical opportunity to identify infected persons and link these inmates with community resources in preparation for their release” [41].

The related value of efficiency is the balance that maximizes outcomes for given resources [1]. For example, there is a need to maximize resources for the benefit of the health of the population:

“In order to effectively design treatment as prevention programs, it is necessary to understand factors associated with HCV transmission so that limited resources can be directed in such a way as to have the largest positive impact through the implementation of public health and treatment as prevention interventions at the population level” [42].

#### Individual vs community interests

Interventions for hepatitis C may favor individual or community interests. Individual interests privilege the right of the individuals to choose how they live their lives, regardless of whether those choices affect their risk of acquiring HCV [27]. Liberalism prioritizes the right of an individual to pursue his or her own conception of good, defined as beliefs about what makes life valuable or worthwhile [27]. For example,

“[t]he illness reality that emerged in interactions with health care practitioners was one that delegitimized participant’s experiential knowledge, priorities and goals in living with hepatitis C as a chronic illness. It also reinforces the authoritarian structures of power that exist with hepatitis C care” [43].

Similarly, libertarianism states that people should have freedom and autonomy of choice so long as it does not interfere with the autonomy and freedoms of others [28]. For example,

“Expanding diagnostic and treatment services is merely a first step in addressing infectious diseases in penitentiaries. A major challenge faced by correctional health care providers is the need to balance individual inmate rights with the health and safety of the wider inmate population” [38].

Autonomy argues for the right for an individual to make his or her own choice [16]. For example,

“[t]he staff becomes legitimized in providing health care that is not at a similar standard to that provided in the broader community. Through this process, the incarcerated women becomes more of an object to manipulate and less of a person in a relationship” [41].

In contrast, privileging community interests suggests that individuals should be held accountable for the community impact of their actions [24]. In other words, decisions to allocate health care resources should account for the life choices of individuals, which may increase their risk of HCV infection [1].

Consequentialism states that the consequences of an action serve as the judgment of the rightness or wrongness of the action [29]. For example, “[u]ntil recently, HCV treatment guidelines in North America categorically excluded illicit drug users from consideration, citing

concerns about adherence, susceptibility for side effects (e.g., depression), and re-infection risks” [44].

Furthermore, although welfarism is a branch of consequentialism, it maintains that individual preferences, desires and decisions are the most important factors when doing an economic analysis [1]. For example,

“The illness realties of participants… points to the need to reconsider the efficacy of the acute care model…this model perpetuates common assumptions about acute illness (e.g. practitioners as expert, disease should be patients top priority) … decontextualized from the patients everyday life and priorities” [43].

Finally, communitarianism emphasizes the responsibility of the individual to the community [24]. The community should be at the forefront of our moral thinking [27]. For example,

“Since they worry about transmitting the infection to others, they notify their injection partners that they are infected…They view HCV infection as a serious disease and make significant effort to avoid sharing equipment …HCV infection is viewed as requiring significant changes in strategies aimed at protecting themselves and others” [45].

## Discussion

Our scoping review on screening, diagnosis and treatment of hepatitis C in Canada addressed CADTH’s goal to incorporate a broader set of social values into HTA [9]. Utilitarian-focused HTA processes may result in inequities in access to screening, diagnosis and treatment programs, because they may disregard important contextual factors specific to vulnerable populations or they may disregard the full social value of the intervention. Further, we identified the diversity of populations affected by hepatitis C and the lack of consensus on how to approach resource allocation decisions. Inclusion of the values, ethics and perspectives of affected populations needs to be considered in designing in HTA decision-making [8]. The following discussion positions our findings on screening, diagnostics and treatment in the literature, followed by a discussion of social values relevant to each study population and study location.

### Screening

In light of access to new DAAs, opinion on the cost effectiveness of HCV screening is divided between (1) those who advocate for widespread access to enable access to necessary services [46], decrease stigma [9, 47, 48], and raise awareness, those who advocate for birth cohort and high-risk population screening, and (2) the Canadian Task Force on Preventive Health Care that recommended no screening for the baby boomer cohort [49]. CADTH suggests that individuals who make the decision to participate in screening programs, take into consideration their life situations and recognition of the stigma associated with screening [50].

Our analysis suggests equity and justice arguments predominate in the literature in support of screening for members of vulnerable populations in addition to some calls for screening for the boomer cohort and those who received blood transfusions prior to 1992. Screening in vulnerable populations seeks to address HCV-related morbidity and mortality [46], both of which are increased if individuals remain unaware of their HCV status and have not been reached by traditional programs due to stigma and lack of trust in health care systems [50–52]. Fear of judgment when interacting with health care providers negates the positive effects of screening [48].

Authors advocate for an equitable approach to screening amongst populations such as PWID, which has the added benefit of enabling access to other social and health services [46]. This body of literature suggests that screening programs be made available without barriers to achieve an equitable approach. Equity arguments are also made with respect to prison populations. While HCV infected prisoners may pose a risk to other prisoners, prisoners also have a right to the same standard of healthcare services provided outside of prison [53].

Further there is support in the literature for a duty to provide screening programs, because it helps raise awareness of HCV, mitigates the spread of infection [47, 48] and provides a public health benefit [47]. It is, therefore, important to address the accessibility of screening services [54].

### Diagnosis

The few articles that discussed diagnosis derived from the HTA literature and analyzed the cost effectiveness of point of care diagnostics, which may reach populations without having to formally engage them in care [55, 56]. Point-of-care diagnostics may aid in reaching vulnerable and geographically isolated populations [56, 57], thereby enhancing equity of access outside of a clinical setting. While diagnosis should lead to the appropriate standard of care, researchers argue that knowledge of disease status is valuable regardless of treatment acquisition [58].

### Treatment

New DAAs are challenging health system budgets in Canada [59], and, to date, there are few implementation guidelines [9]. DAAs provide greater sustained virological response and fewer side effects [48], however, access for many populations remains limited [50, 60]. Some studies consider that the prevention of worsening disease and reduction in the need for invasive procedures offsets the initial high cost of treatment [61]. However, this approach in a large population has significant budget impact, creating substantial opportunity costs for the funding for other health care services [9, 61]. As a result, Canada has adopted a model that prioritizes those with more severe disease (fibrosis score greater than 2 on a 4-point scale) [61], but this approach does not capture the proposed benefits of treatment as a means of preventing transmission, a strategy that requires further cost-effective analyses [62].

The literature on high-cost therapies focused on efficiency and population benefit and recognized the high patient demand for treatment, but there was no consensus on which population should be prioritized for treatment [63]. Some stipulated that treatment management is necessary to ensure cost-effectiveness in combination with treatment prioritization for high-risk populations [64]. Some discussed social values with respect to access to medication for marginalized populations, which remains problematic in Canada’s health care systems [65].

### Population specific considerations

A range of social values was evident in the literature on HCV screening, diagnosis and treatment for both vulnerable and non-vulnerable populations.

PWID are the population at the greatest risk for acquiring HCV [50, 66], raising issues of a duty to provide care with obvious population health benefits [50, 66]. Indeed, “the time has come for a targeted and proactive HCV treatment approach for [PWID], and that it is feasible and desirable from a public health perspective” [67]. A targeted suite of HCV-related services for PWID would both meet the needs of a large and vulnerable population and reduce the transmission of HCV [67–69]. A community based and multisectoral approach to treating HCV amongst PWID would tackle more than just the clinical effects of the disease; it would positively impact the social determinants of health, by connecting PWID with a broader range of services [70]. Specifically, targeted screening services might enable healthcare providers to address both physical and psychological concerns and connect PWID to other services [12, 47, 64, 71].

Authors concluded that a targeted approach to screening, diagnosis and treatment of HCV would reduce inequities through provision of accessible, effective care. However, social stigma of drug use creates inequities in healthcare. PWID are faced with the bias of health care professionals in terms of willingness to provide high cost therapies [44, 70], though studies have found similar sustained virologic responses [46]. Practioners and institutional structures reinforce stigmatization of PWID patients; their symptoms are commonly discounted or PWID are underserviced [43]. Such stigmatization of PWID may lead to the devaluation of persons, which transforms HCV from a health issue into a moral one [72]. A resultant and societal belief is that PWID are less deserving of care than other patients, because their HCV is perceived as the result of self-inflicted cause [43].

Social values with respect to prison populations primarily fell in the categories of equity and justice and the duty to provide care. An example of the latter derives from Canadian Correctional Services (CSC), which stipulates that inmates are owed access to health care services and should not have different outcomes due to imprisonment [40, 53, 73]. Numerous authors suggested that a targeted treatment program for HCV positive members of the prison population would be just and economical [63, 67]. It would provide treatment to an overlooked population with a disproportionate rate of HCV infection, members of which might not otherwise seek treatment [40, 53], thereby maximizing population benefit by decreasing transmission both within and outside prisons [40]. However, the provision of health care services for this population remains largely insufficient, with the needs of individuals not being met or met with significant barriers to access [74].

This lack of access is contrary to the guarantees for universal, comprehensive and accessible health care in the *Canada Health Act*. There is a culture of depersonalization within the prison system, whereby prisoners are not seen as persons but more so as “permanent criminals”, who are not prioritized for screening [53] or treatment [74]. Such depersonalized treatment results in prisoners being treated as less deserving than other populations [74]; they are neither provided with adequate care nor treated equitably with the same standards of care as non-prison populations [53].

The few articles that specifically addressed HCV in indigenous populations focused on issues of equity and justice, reflecting the historical injustices and systemic oppression faced by indigenous people in Canada and structural factors [75]. Indeed, HCV infection is a product of substance use and other risk behaviours that are themselves precipitated by trauma and racism relating to colonization and residential schooling [76]. Trauma and disease should therefore be examined together in order to determine the most appropriate course of action [77]. Authors advocated for increased multi-level approaches, with interventions tailored to address specific needs through incorporation of culturally safe approaches [33]. Authors recommended that interventions be developed and implemented in partnership with community members [77] and address family, community, environmental and cultural factors [77, 78].

In contrast to vulnerable populations, literature on the baby boomer cohort, born between 1945 and 1975, focused on the maximization of population benefit. Baby boomers have disproportionate rates of HCV and the provision of DAAs would reduce prevalence of HCV and associated complications [64]. Complications include hepatocellular carcinoma, which requires a liver transplant and equates to high healthcare utilization. Addressing HCV in this population might therefore reduce health care expenditures and maximize population benefits [64], because the cost of treatment is approximately $80,000 compared to $104,000 for a liver transplant, not including antirejection medication and follow up care [64]. Some authors called for age cohort screening to capture a large number of active HCV infections [35], which might prove cost effective, despite high up-front costs [12], in contrast to the Canadian Task Force on Preventive Health Care (2017) determination of insufficient cost-effectiveness evidence to support age cohort screening [22].

Equity and justice claims were, however, made with respect to the sub-population of baby boomers who were infected as the result of a contaminated blood transfusion [79]. Targeted look back programs are in place to notify blood and blood product recipients of potential health issues arising from their previous transfusions. These programs, together with compensation programs, represent justice for recipients of contaminated blood and blood products [79]. Furthering justice claims, the Krever Commission or Krever Inquiry- Inquiry on the Blood System in Canada [80] recommended that all patients who had received a blood transfusion between 1978 and 1990 should be identified to provide them with necessary medical care [81]. Additionally, Canada has since provided educational and financial support to HCV positive individuals in this category and encourages other countries to follow suit [82, 83]. In contrast to vulnerable populations, Canada set aside $1.1 billion dollars to compensate individuals who received contaminated blood, because they suffered a negative “no fault” health outcome [84].

### Study location

Of the minority of articles that specified a study location, the majority were conducted in urban and inner city locations. These locations have the highest population density of HCV-infected individuals [50]. However, focus on these locations results in an evidence gap for rural and remote locations [85]. Populations in these locations require special consideration for HCV screening, diagnosis and treatment due to geographic isolation and inequities in healthcare services and delivers. More resources are therefore required for rural populations, especially with respect to education and risk communication [86]. Coordinated outreach teams might provide screening, counseling and treatment for people living outside of the city limits to ensure availability and accessibility of care [86] and improve trust between physicians and patients to encourage people living away from medical centres to seek care [86]. Rural patients face long wait times to see specialists, most travel further distances to major medical facilities and take more time off work, constituting an opportunity cost [85]. This is amplified for indigenous populations living in rural and remote settings [31]; many go undiagnosed and have difficulty accessing screening and treatment [87]. Authors concluded that persons who are geographically isolated should be prioritized for HCV outreach programs.

### Limitations

Our study may not have identified the full set of social values. Our qualitative analysis of social values was based on an a priori coding frame, which may not have included all possibly relevant social values. The distribution of studies reflected in the academic literature on hepatitis C in a Canadian context may have further contributed to a narrowing of the range of social values identified. For example, studies rarely addressed issues affecting rural and remote communities and specific sub-populations, such as at-risk youth. Potential coding errors were mitigated through double coding, wherein 2 coders independently evaluated the same set of articles. This achieved a 0.85 to 0.95 kappa score indicating strong agreement. Disagreements were resolved by consensus before independently coding the remaining articles.

## Conclusion

Our study suggests that the academic literature may be used to identify an expanded set of social values to be taken into account by resource allocation decision makers in financially constrained environments. Our analysis of hepatitis C screening, diagnosis and treatment in the context of curative, but high cost and large budget impact treatment options highlighted social values specific to vulnerable populations, which may augment the predominantly utilitarian calculus applied in most HTAs. Authors clearly call for greater consideration of equity and justice and the duty to provide care in making evidence-based recommendations for screening, diagnosis and treatment of different populations and in different settings that account for consideration of individual and community interests. These values particularly address social determinants of health as well as specific needs and access for the marginalized populations that are most at risk of HCV infection. Specifically, authors call for more tailored approaches to screening, diagnosis and treatment of HCV that considered the needs and life circumstances of different populations in different settings. Such resource allocation decision-making would be facilitated through the implementation of dialogic processes that are inclusive of the views of affected populations.

## Data Availability

The full list of publications reviewed is available from the authors, however, the dataset of academic articles cannot be deposited due to copyright restrictions.

## Abbreviations

CADTH: Canadian Agency for Drugs and Technology in Health
DAA: Direct Acting Antivirals
HCV: Hepatitis C Virus
HIV: Human Immunodeficiency Virus
HTA: Health Technology Assessment
PWID: People who Inject Drugs
